# Climate conscious pharmacy practice: a qualitative interview study with pharmacists in the context of respiratory health care

**DOI:** 10.1007/s11096-025-02005-y

**Published:** 2025-09-23

**Authors:** Rabia Cameron, Yasir Alhazmi, Philip Chi Lip Kwok, Bandana Saini

**Affiliations:** 1 Sydney Pharmacy School, Faculty of Medicine and Health, University of Sydney, A15, Science Rd, Camperdown, NSW 2050 Australia; 2 Department of Clinical Pharmacy, College of Pharmacy, Najran University, 64462 Najran, Saudi Arabia; 3 Woolcock Institute of Medical Research Sydney, 2 Innovation Rd, Macquarie Park, NSW 2113 Australia

**Keywords:** Asthma, Chronic obstructive pulmonary disease, Climate change, Pharmacists

## Abstract

**Introduction:**

Climate change negatively impacts millions of people with respiratory conditions. On the other hand, respiratory health care negatively impacts climate change given that mainstay inhaler treatment poses its own environmental risk. Pharmacists are frequently involved in managing chronic respiratory conditions including counselling on inhaler use, thus they are well-positioned to reduce associated environmental impacts, however this role is unexplored.

**Aim:**

This study aimed to investigate Australian pharmacists’ perceptions on the impact of climate change on respiratory health and the impact of respiratory health care provision or treatment use on the environment. We also aimed to explore pharmacists’ views about their potential roles in promoting sustainable respiratory health care.

**Method:**

Following approval from an institutional ethics review committee, qualitative semi-structured interviews were conducted with consenting pharmacists who had at least one year of post-registration experience and were currently working in any clinical setting. The interviews explored pharmacists’ general perspectives on climate change before delving specifically into respiratory health care. Interviews were recorded, transcribed verbatim and subjected to an inductive thematic analysis within a constructivist paradigm.

**Results:**

Thirty-two participants (72% female, 28% male) were interviewed. Three key themes were derived from the analysis: (1) environment considerations as an afterthought, (2) linking environment to respiratory care, and (3) working towards sustainable practice. Patient health was expressed as the main concern in clinical practice, with environmental considerations reported as lower priority. Most participants saw their role as being the management of patients ‘respiratory symptom control’. Barriers to climate action described by participants included prescriber and patient acceptance of recommendations and concerns about counselling patients on switching to low carbon footprint inhalers being beyond their current scope of practice. Participants recommended multi-stakeholder collaboration and inclusion of this topic in pharmacy curricula as key factors to address to build sustainable respiratory health care provision.

**Conclusion:**

Pharmacists’ main focus in respiratory health care provision is on clinical rather than environmental change issues. Many barriers such as concerns about patients’ acceptance of sustainability related advice or counselling, and limited training on the topic were cited. Further research should explore best ways to address these issues, preferably in co-design practices with stakeholders.

**Supplementary Information:**

The online version contains supplementary material available at 10.1007/s11096-025-02005-y.

## Impact statements

- General awareness about or practice change in pharmacy practice regarding sustainable respiratory health care is still low.
- Pharmacists recognise a role in facilitating the sustainable use of inhalers, but several barriers appear to inhibit substantial practice change.
- Embedding sustainable practice training in pharmacy education is important to support practice change.
- Professional guidelines and incentives are needed for pharmacists to practice sustainability and advocate for sustainable use of medicines.

## Introduction

Chronic respiratory conditions including asthma and chronic obstructive pulmonary disease (COPD) impact millions of people worldwide [1, 2]. Climate change, defined by the United Nations as *‘long-term shifts in temperatures and weather patterns’*, can adversely affect those with respiratory conditions [3]. Numerous climate change-related lung effects are driven by extreme temperature exposures and changes in air quality related to concentrations of particulate matter, ozone and aeroallergens [4]. Particulate matter smaller than 2.5 microns (PM_2.5_) can accumulate in the lower airways causing epigenetic modifications that disrupt the immune response and exacerbate airway inflammation [4]. Ozone, being a reactive species, can trigger oxidative damage across cells within the lung [5]. Furthermore, changed weather patterns can alter pollen seasons and pollen allergenicity which can increase the duration of symptoms [6, 7]. Temperature extremes, particularly higher temperatures, can alter lung functions through inflammatory changes, enzyme induction and oxidative damage [8]. Changed weather patterns can also catalyse antimicrobial resistance, making infective exacerbations difficult to manage [9].

On the other hand, respiratory healthcare has a significant negative impact on climate change [10]. Inhalers form the mainstay of treatment in asthma and COPD. These include pressurised metered dose inhalers (pMDIs), dry powder inhalers (DPIs) and soft mist inhalers (SMIs) [11]. All three device types contribute negatively to the environment throughout their lifecycle stages: manufacturing, transportation, use and disposal [10]. However, pMDIs incur an additional environmental cost since the hydrofluorocarbon propellants in their formulation, being potent greenhouse gases, have a high global warming potential [12]. Additionally, respiratory exacerbations requiring step-up treatment, doctor visits or hospitalisations impose a considerable environmental cost [13].

Australia, as a case in point, has a high prevalence of asthma and COPD [14]. Healthcare contributes to 7% of Australia’s total carbon footprint; 19% of this amount can be ascribed to pharmaceuticals [15]. Aerosol inhalers (pMDIs) alone contribute to 1.7% of Australia’s healthcare carbon footprint [15]. From a health professional perspective, key directives to manage the two-way impact of climate change on respiratory health and health care would include environmentally conscious inhaler use, improved respiratory symptom control and self-management education [16]. Guidelines now suggest that if equivalent symptom control is achievable, then bearing in mind the patients' inhaler use skills, device preference and device availability pMDIs could be substituted with lower carbon footprint inhalers such as DPIs [10]. To implement these guidelines, all health professionals involved may need to modify their health care delivery approaches for patients with chronic respiratory conditions.

Community pharmacists are one the most accessed professionals in Australia; one consumer visits a pharmacy at least 18 times a year on average [17]. Robust evidence demonstrates that Australian pharmacists can play an effective role in chronic respiratory disease management [18–20]. However, there is a paucity of research exploring *if* and *how* this role in respiratory health care incorporates environmental sustainability alongside patient health goals. Given that pharmacists are frequently involved in the facilitation of quality use of respiratory medicines, this is a conspicuous starting point in a planned reduction of the Australian respiratory care carbon footprint.

### Aim

This study aimed to investigate Australian pharmacists’ perceptions on the impact of climate change on respiratory health and the impact of respiratory health care provision or treatment use on the environment. We also aimed to explore pharmacists’ views about their potential roles in promoting sustainable respiratory health care.

## Method

### Study design

Given the exploratory nature of the study, a qualitative method utilising semi-structured interviews for data collection was selected [21].

### Participant inclusion/exclusion criteria

Eligibility criteria included pharmacists on the Australian Health Practitioner Regulation Agency (Ahpra) registry at the time of the study with at least one year of post-registration practice experience and currently undertaking work in any clinical setting (e.g. community, consultant practice, hospital). Pharmacists with provisional registration or not currently practicing were excluded from the study. The sample size plan was based on the concept of data saturation, i.e. the point where data being gathered repeats what previously gathered data already suggests [22].

### Recruitment and sampling

**Fig. 1:**
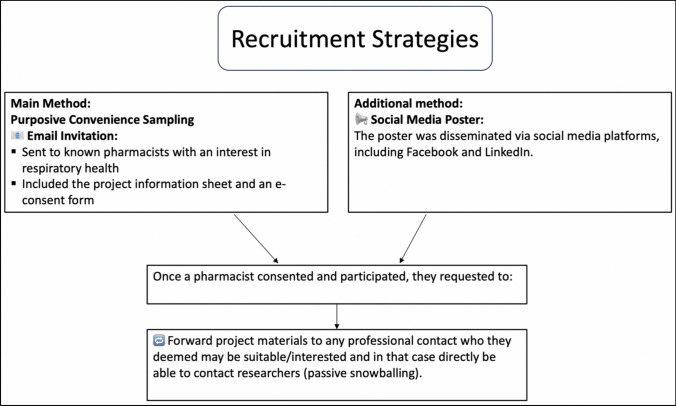
Recruitment strategies

Participants were recruited using purposive convenience sampling by first inviting primary contacts of the researchers and subsequently via passive snowballing (peer-nomination). A recruitment poster was also disseminated via social media on Facebook and LinkedIn (Fig. 1). Informed consent was obtained from participants using an electronic REDCap form (Research Electronic Data Capture, which is a secure, web-based application).

### Data generation

Semi-structured interviews were conducted by one researcher (RC) online via the Zoom platform (Zoom Video Communications, Inc., 2024) between August and September 2024. Interviews were audio-recorded, transcribed verbatim and proofread against the recording. An interview guide (Supplementary material), based on a review of the literature and the clinical and science-based expertise of researchers, assisted in the interview process. It was piloted with four pharmacist colleagues before use with participants. The interview guide was cognitively funnelled, with initial rapport building and straightforward questions followed by more thought-provoking questions towards the end [23]. For example, towards the end of the interview, participants were presented with a hypothetical scenario of a patient prescribed a pMDI and, given that a clinically equivalent DPI was available, asked for their clinical recommendation. Participants’ demographic details were collected at the end of the interview.

Recordings and live closed captioning data files were downloaded from the Zoom interface and stored in a secure University server space. Once transcription quality was verified to be accurate and verbatim, transcripts were de-identified. The coding key associated with each participant was destroyed rendering the data unidentifiable henceforth.

### Analysis

All transcripts were subjected to inductive thematic analysis using the NVivo 14 software for data management. Analysis followed Braun and Clarke’s Six Step data analysis process which required careful in-depth reading to source meanings, attributing codes, developing a coding hierarchy based on seven initial transcripts, coalescing codes into overarching themes, reviewing themes across other transcripts until thematic saturation was acquired, renaming themes, and producing the report [24, 25]. Analyses were undertaken primarily by RC with an epistemological stance of constructivism and an ontological position of realism [26, 27]. Independent coding of 20% of the transcripts was undertaken by two authors to validate analyses by building a shared team meaning of the data. The analysis and reporting of the research followed the COREQ guidelines to assure rigour [28].

### Reflexivity

The interviewer and primary data analyst (RC) in this study was a female pharmacy honours student who has a personal pro-sustainability position which may have biased her in over-emphasising similar themes as they emerged from the data. However, a proportion of transcripts were independently analysed by more senior researchers (BS) and experienced pharmacists of another gender (YA and PK) to mitigate these biases. The overall awareness of evidence around environmental harms of inhalers may have also influenced the team towards a pro-sustainability depiction in the results.

### Ethics approval

This study was granted approval (2024/HE000672) by the Human Research Ethics Committee (HREC) of The University of Sydney.

## Results

A total of 32 participants were interviewed in August and September 2024 and their demographic details recorded (Table 1); all interviews were conducted online via Zoom. Of these participants, 28% identified as males and 72% as females. The participants were distributed across several Australian states. Three major themes were developed: (1) environment considerations as an afterthought, (2) linking environment to respiratory care and (3) working towards sustainable practice. Exemplar quotations are added to the text to showcase theme derivation; additional details on subthemes and codes with supporting quotations are also fully outlined in Tables 2, 3, and 4 (Supplementary material).

**Table 1:** Demographic characteristics of participants attending qualitative interviews

Demographics	N = 32	
	*n*	%^
Gender		
Female	23	72
Male	9	28
Age bracket		
22–34	17	53.1
35–44	8	25
45–54	3	9.3
55–64	3	9.3
65+	1	3.1
Total years of experience as registered pharmacist		
1–9	16	50
10–19	8	25
20–29	4	12.5
30–39	3	9.3
40–49	1	3.1
State		
New South Wales	21	65.6
Queensland	4	12.5
Victoria	4	12.5
Western Australia	2	6.2
South Australia	1	3.1
Current pharmacy practice type*		
Community independent	7	–
Community banner	16	–
Hospital	5	–
Academia	11	–
Consultancy	3	–
Regulatory	1	–
Governance	1	–
Health professional training degree(s)*		
Bachelor of Pharmacy	31	–
Bachelor of Medical Science	1	–
Graduate Certificate in Pharmacy Practice	5	–
Graduate Diploma in Clinical Pharmacy	2	–
Graduate Certificate in Sleep Medicine	1	–
Graduate Certificate in Clinical Pharmacy	1	–
Aged Care		
Master of Pharmacy	1	–
Master of Philosophy and Pharmacy	1	–
Master of Public Health	1	–
PhD in Pharmacy Practice	2	–
Previous training in environmental health		
Yes	2	6.2
No	30	93.7
Previous environmental-related advocacy work		
Yes	4	12.5
No	28	87.5
Organisation’s specific environmental goals		
Yes	5	15.6
No	16	50
Unsure	11	34.3

Where N is the total sample size and n is a subset of total sample size based on frequency

^*^Options within the demographic group are not mutually exclusive

^Percentages calculated within each demographic group

### Environment considerations as an afterthought

#### Relevant yet minimal consideration

Climate change, though considered important by all participants, was not prominently referenced in discussions on health care provision. Some participants expressed that climate change was relevant to their practice, for example, in being a trigger for respiratory symptoms. However, a few participants perceived climate change as playing no direct role in their practice and indicated this was their reason for not focussing on environmental consciousness in their pharmacies.

“I don't know how often we think about it since a lot of times we're so focused on just doing our clinical work, that we may not think about those little things even though we say ‘little’ it's not really a little issue. It is a big issue, just something that we may not focus as much on.” (participant #27)

#### Patient health prioritisation

Most participants expressed that patient health was the main concern in clinical practice, ranking environmental consideration relatively low in priority. Given this prioritisation, some of these participants emphasised that therefore there would be no value in integrating environment-related discussions in their counselling. A few participants acknowledged that their indifference towards sustainable practice stemmed from a lack of environmental sustainability training in pre-registration years, given their training had focussed on clinical topics only. Participants generally prioritised patient health and patient inhaler usage skills or preference above all other considerations when posed with the hypothetical scenario regarding choice of inhaler type [the hypothetical scenario is fully described in the interview guide (Supplementary material)].

“It’s very difficult to counsel patients about the environment because no offence but realistically speaking not many people care too much. I probably wouldn’t because clinically it doesn’t matter.” (participant #16)

#### Uncoordinated efforts

Participants commented on the ‘disconnect’ of the efforts around sustainability between governmental decisions, healthcare policies, the pharmacy profession and individual pharmacists. Most felt that the pharmacy professions’ attempts to advocate for sustainable practice were neither strong nor sufficient. Sustainable actions were reportedly often performed on an individual scale, driven through personal motivation by a few participants or their colleagues and included general actions such as their pharmacy waste disposal. Some pharmacists professed making minimal contributions towards sustainability in their pharmacies, describing sustainable practice concepts as ‘*negligible in the grand scheme of things*’.

“I don't know how well-versed our profession is in terms of what the actual impact is on climate change on respiratory health and vice versa, in terms of even the use of inhalers.” (participant #27)

### Linking environment to respiratory care

#### Environment and respiratory advice

Participants stated their primary, and sometimes only, role in minimising the environmental impact of inhalers was in managing respiratory control through counselling about inhaler technique or adherence. Some participants indicated that they actively informed patients about dose counters on inhalers to ensure full consumption, as well as safe disposal. Most participants spoke positively about reusable soft mist inhalers as an example of environmentally friendly treatment; however, one participant expressed the opinion that even switching people to these inhalers would make insignificant contributions towards reducing greenhouse gas emissions. When asked about counselling patients during climatic events, only a few participants provided examples tailored to specific weather conditions, such as mould eradication post-flooding. Most participants did not provide in-depth patient education around climate change impacts on respiratory health—highlighting only general respiratory care points like trigger avoidance and inhaler use when probed on the topic. Several participants, however, mentioned that the community pharmacy setting is most conducive towards inhaler-related or even environmental management advice due to high-frequency patient contact.

“I think everyone agrees that the most environmentally impactful thing that we can do is better control of asthma.” (participant #24)

“Explaining to patients, do not throw things into landfill, but to come bring it back to the pharmacies to be put into the return unwanted medicine (RUM) bin, so they can be incinerated is a big awareness that needs to be done.” (participant #29)

Notably, the return unwanted medicine (RUM) project involves the provision of medicine disposal bins to all pharmacies in Australia with bin collection scheduled by pharmacies when these bins are full. The medicine contents in collected RUM bins are examined by experts and disposed of in the safest manner [29].

#### Barriers to change

Participants outlined a wide range of limitations which hindered sustainable pharmacy practice. Many participants felt that they made minimal contributions to environmentally responsible respiratory health care provision.

“I didn't know I had a role at all. Maybe this is a helpless mindset, but I don't know how else to advocate to not have so much product from the inhalers.” (participant #25)

Most participants felt that their role was limited to advising patients about medicine disposal. Several participants were uncertain about being able to predict environmental events which may cause respiratory flareups in patients. Additionally, not being able to assure patients about the continued supply of essential medications during situations such as emergency evacuations during bushfires or floods, also left participants feeling powerless. Finally, although participants noted that patients reported that environmental triggers were associated with their respiratory exacerbations, there was not much evidence based advise pharmacists could provide, apart from recommending patients adhere to medicines and follow doctor provided action plans. These situations left participants less satisfied about their ability to help patients.

Interestingly, one participant who had asthma themselves empathised with this issue, as their lived experience provided them with an insight into patients’ challenges around managing asthma in difficult environments.

Nearly all participants self-assessed that there was a broad gap in their knowledge around sustainability relating to pharmacy practice and respiratory health care. Some felt minimal sustainable actions in pharmacy practice stemmed from a lack of guidance and resources compared to, say, at a domestic level where local councils facilitate recycling bins or provide incentives for using solar panels. Only a few participants were able to recognise that pMDIs are more environmentally harmful than other inhaler types. However, one noted that there is hesitancy in relaying this information to patients without sufficient knowledge themselves.

“To be honest, I think I'm at that stage where it's more about the awareness. Not knowing exactly what to do in our practice, at least in the pharmacy-specific environment. I mean, at home we talk about recycling and electronic [Electric] vehicles.” (participant #1)

Participants expressed that limited time and the focussed responsibility as a pharmacist, such as dispensing and managing pharmacy services, are barriers when considering a switch to environmentally friendly inhalers. Some participants suggested a likely future role in simple inhaler switches. However, several obstacles deter this scope of practice: inaccessibility to patient medical records, a restriction in prescribing rights for inhaler switching, and a lack of remuneration for such a service.

“That will save my time. As a pharmacist, I've got so much in my plate. If I had to ring the doctor each time for scripts, let's be honest, no matter how good is for the environment it will deter you from doing that right?” (participant #10)

There were some interesting discussions about the delegation of authority around patient-related clinical decision making. Participating pharmacists perceived that physicians (general practitioners or specialists) have greater input into patient-related decisions and hence switching to an environmentally friendly inhaler should occur at this stage. Hesitancy in calling the general practitioner with a request to change an inhaler merely from an environmental cost viewpoint was not felt to be a valid reason and uncertainty of a physician giving credence to and responding favourably to their suggestion rendered many hesitant to undertake this chore.

“I wouldn't take any action. It's a prescriber's choice as to what medication to prescribe. Also, the familiarity most patients have with the typical metered dose inhalers may improve compliance.” (participant #15)

Organisational policies were another impediment. For example, participants observed that while there are policies on the disposal of medicines (inhalers); these policies may not be adhered to, as they often incur costs for the pharmacy business. For example, participants reported that recycling options required to safely dispose of blister packs used in dose administration aids were costly.

“I think we looked into trying to organise a blister pack recycling bin for the pharmacy. But that involves money. And so we ultimately didn't get a final say in that.” (participant #2)

### Working towards sustainable practice

#### Desire for change

Participants recognised that climate change is a pressing topic that requires significant attention to build sustainable health care and improve respiratory health. There was a recognition for the impact a *‘domino effect’* would have where motivated peers could promote action for impactful changes. Indeed, individual will was demonstrable as several participants described undertaking or exploring initiatives they could action in their workplace, for example investigating disposal services. Though reticent, many participants declared a willingness, given enough sufficient training, to balance ‘patient outcomes’ and ‘environmental concerns’ in recommending inhaler switching when clinically equivalent, lower environmental impact inhalers were available. In some cases, patient requests about the environment may serve to catalyse this thinking. There were no observable differences in coding frequency between transcripts except that female participants more strongly expressed pro-environmental behaviours.

“And similarly with organisations within hospitals, when we see one place doing one thing then there could be that domino effect, then we can benefit from improving respiratory health and improving climate change.” (participant #27)

#### Need for leadership from larger organisations

Many participants recognised the importance of a multi-stakeholder drive to change practice. Government, policy makers, manufacturers and pharmaceutical professional bodies were seen collectively as key stakeholders equipped to innovate change and disseminate regulations where individual participant action may be ineffective or insufficient to make a sizeable difference.

“I think I could have a key role in that but that role would be predicated on key support from correct stakeholders.” (participant #2)

Suggestions for positive roles that stakeholders can implement in the future were made by participants. Participants recognised policy mandates on plastics in other industries and suggested a similar approach to be adopted with single-use inhalers without impacting patient safety. A large onus, according to many participants, lay with pharmaceutical companies in adopting green processes in manufacture and distribution, environmentally friendly inhaler products, transparent recommendations about reusability as well as inhaler disposal services. Pharmaceutical professional bodies were cited as drivers of change through continuing professional education and standards for sustainable pharmacy practice. Many participants stated that pragmatic guidelines on sustainable practice needed development and dissemination.

“But it's gonna go to the back of our head about the environment if there isn't some policies in place to promote us to do that.” (participant #25)

“So, the manufacturer definitely needs to play a part because at the end of the day the bottom line is they are the people who invented this device.” (participant #23)

“So, if those could be embedded into the lecture as well as the current resources by the peak bodies. Educational resources that be helpful.” (participant #9)

It was felt that larger pharmacies had wider networks to incite change (e.g. convert to energy-saving lights across each business unit). Research institutions were seen by participants as having substantial power to propel change through practice research and generation of evidence for environmentally friendly inhaler products. Most participants voiced the need for remuneration with services centred around sustainable health care to compensate time spent, without which change in practice was unlikely.

“Initially there might be slow uptake then traction… then state-wide or national scale there is a drastic impact on the environment. There are 3500 pharmacies in Australia? If everyone does it because pharmaceutical companies might pay or the government has incentives, you might have a sizeable impact on the environment. Otherwise, no one will do it.” (participant #16)

## Discussion

### Statement of key findings

This is the first Australian study to explore pharmacists’ perspectives on climate change, its impact on respiratory health and the pharmacist role in sustainable respiratory health care. Participants believed that climate change was an important issue and that pharmacists had the potential to contribute towards carbon footprint reductions in respiratory care. However, most expressed that patient health was the primary concern in clinical practice, ranking environmental considerations relatively low in priority. Discussions were offered about personal, professional and systemic barriers when tackling sustainability in respiratory health. Despite increasing literature on climate change impacts affecting respiratory health and vice versa, there appears to be minimal awareness and change translated in pharmacy practice and healthcare at large—this issue needs urgent action.

### Interpretation

Participating pharmacists, being clinical practitioners, focused on ‘clinical’ outcomes rather than on environmental sustainability. Although participating pharmacists expressed a lack of awareness about the reciprocal impact of climate change on respiratory health, they provided examples of clinical actions that reduce the carbon footprint of respiratory health care. For example, patient education on medication adherence and inhaler technique reduces disease burden by improving symptom control, thereby lowering health care resource consumption. Many participants hesitated to counsel patients on how their treatment impacts the environment or vice versa. This hesitancy may arise from being uncertain about appropriate actions to recommend, suggesting that training at the pre-registration level on the management of health impacts of climate change is critical. Indeed, participants themselves expressed a need for more training around sustainable practice. In such training, a clear strategy could be to emphasise that delivering respiratory care based on national guidelines and evidence-based service models [18–20] can improve clinical and economic outcomes and enhance sustainability in respiratory health care [30].

Nevertheless, many participants were hesitant about counselling patients about the environmental impact of inhalers given uncertainty about appropriate actions to recommend. Although there is limited research on this specific area, a qualitative study in the UK explored the views of primary care health professionals, including pharmacists, and reported that the lack of pharmacist training was evident [31]. From a general climate consciousness viewpoint, our results suggesting reticence about roles in sustainable care provision are consistent with findings from another study conducted with community pharmacists highlighting the importance of including climate-related topics in the pharmacy curricula [32]. Furthermore, a recent systematic review highlighted that hospital pharmacists enacted lead roles in sustainable practice around waste disposal of medicines and sustainability-related decision making, though reviewed studies were open to methodological bias [33]. Our participants were mainly community pharmacists and noted that larger pharmacies were better equipped for sustainability. Hospital pharmacy practice may have an advantage in having systemic frameworks, reflected in literature from this sector [33]. The key message is a need for curricular and professional development campaigns around sustainable practice for pharmacy students and pharmacists.

Our results also highlighted that participants did not feel equipped to advise patients on managing respiratory health during climate conditions. Given Australia’s high prevalence of chronic respiratory conditions and extreme weather conditions (e.g. bushfires, floods), this ‘gap’ is noteworthy [34–36]. When presented with a hypothetical scenario about weighing the environmental impact of inhalers when potentially recommending a switch, participants prioritised patient inhaler preference and use over environmental impact. This is consistent with recent research that indicates healthcare professionals, including pharmacists, prioritise clinical efficacy, ease of use, and patient adherence over environmental considerations when selecting inhalers [37]. This view held by many of our participants is concordant with guidelines which suggest that the ‘greenest inhaler’ is one that can be used by patients correctly, but our participants were hesitant to switch to lower carbon footprint inhalers in general. Therefore, this is an important issue to address [10].

There is significance in pMDIs contributing to Australia’s healthcare carbon footprint. Short acting beta agonists (SABAs) are available without a prescription in Australia and mostly used in the form of pMDIs. These are often sourced by patients given their accessibility, as patient do not require a medical consultation for a prescription. However, SABA over-reliance is a serious risk for poor asthma outcomes [38]. Environmental advocacy to substitute SABA-containing pMDIs with anti-inflammatory relievers (AIRs) in a DPI form may derive triple benefits—clinical, environmental and economic [39]. Notably, AIR inhalers include a low dose corticosteroid as an anti-inflammatory treatment as well as a quick onset but longer acting beta agonist which serves as a bronchodilator. Studies indicate that patients with respiratory conditions are willing to switch to low carbon footprint inhalers—pharmacists therefore need to align their practice accordingly in a patient-centred approach [40]. Hence, to resolve participants’ concerns on recommending environmentally informed inhaler choices to physicians, interprofessional education and research on implementation for an ‘across-the-board’ adoption is needed.

Participants were cognisant that climate change was too big an issue for any one group to tackle. They made multiple suggestions for change at various levels, including the need for multi-stakeholder collaboration. At the *macro* level, legislated carbon targets for different industry sectors may be useful in Australia or globally [41]. Examples from other countries could be recommended, e.g. the UK Government and the NHS’s legislated carbon targets (although research suggests that adoption of these targets has not reached pharmacy departments in public hospitals) [42]. Further integration or incentivisation of sustainability measures in workplaces were also suggested by our participants, along with clear frameworks and guidelines. Perhaps reimbursement for interventions reducing pharmaceuticals’ carbon footprints could help [43, 44]. The pharmaceutical industry also needs to accelerate the development of environmentally friendly inhalers. At the *micro* or personal level, advocacy stemming from individual practitioners may build momentum for change, e.g., these individuals could be showcased as role models to prompt other pharmacy practitioners to not simply ‘delegate’ decisions around inhaler use to physicians [32, 46].

### Strengths and weaknesses

While there were participants classified into each age group, they were not distributed evenly which limits perspectives from pharmacists in older age groups. Younger participants may have more awareness about sustainability [45] Further, most participants in our study were female—although this is representative of the profession where females make up 64% of the Australian pharmacist workforce [47], hence the results are transferable in this context. We note however that gender may influence sustainability perspectives, therefore we may not have captured perspectives of male pharmacists adequately in this study [48]. Interviewing bias may have affected how participants responded to questions on climate change to the interviewer. However, the developed interview guide was uniformly used in all interviews.

Regarding trustworthiness, our independent review of transcripts, audit trail review and reflexivity enhances the conformability and dependability of the analyses, resonance with prior relevant research suggests dependability, and the thick descriptions shared in tabulated data offer transferability [49].

### Further research

While there is a lack of awareness among pharmacists regarding the reciprocal relationship between pharmacy practice and climate change among pharmacists, Further research should explore best ways to address these issues, perhaps in co-design practices with stakeholders. Additionally, research is required to develop non-pharmacological guidelines to assist pharmacists in managing respiratory diseases during extreme weather conditions.

## Conclusion

This study identified the current context of pharmacy practice regarding climate change issues with respiratory health. Pharmacists’ main focus in respiratory health care provision is on clinical rather than environmental change issues. Many barriers such as concerns about acceptance of sustainability related inhaler selection advice or counselling of patients, and limited training on the topic were cited. Future research should explore the perspectives of other healthcare professionals, such as physicians, or co-design practices with pharmacists and other stakeholders.

## Supplementary Information

Below is the link to the electronic supplementary material.

Supplementary file 1 (DOCX 620 KB)

## Data Availability

Due to the nature of the interviews, respondents were assured raw data would remain confidential and would not be shared.
