# Supplementary material for: Climate conscious pharmacy practice: a qualitative interview study with pharmacists in the context of respiratory health care
Source: Int J Clin Pharm. 2025 Sep 23;48(2):490–500. doi: 10.1007/s11096-025-02005-y (PMC12992453; doi:10.1007/s11096-025-02005-y)
Supplement: Supplementary file 1 — (DOCX 620 KB) [file 11096_2025_2005_MOESM1_ESM.docx]

**Interview guide**

**
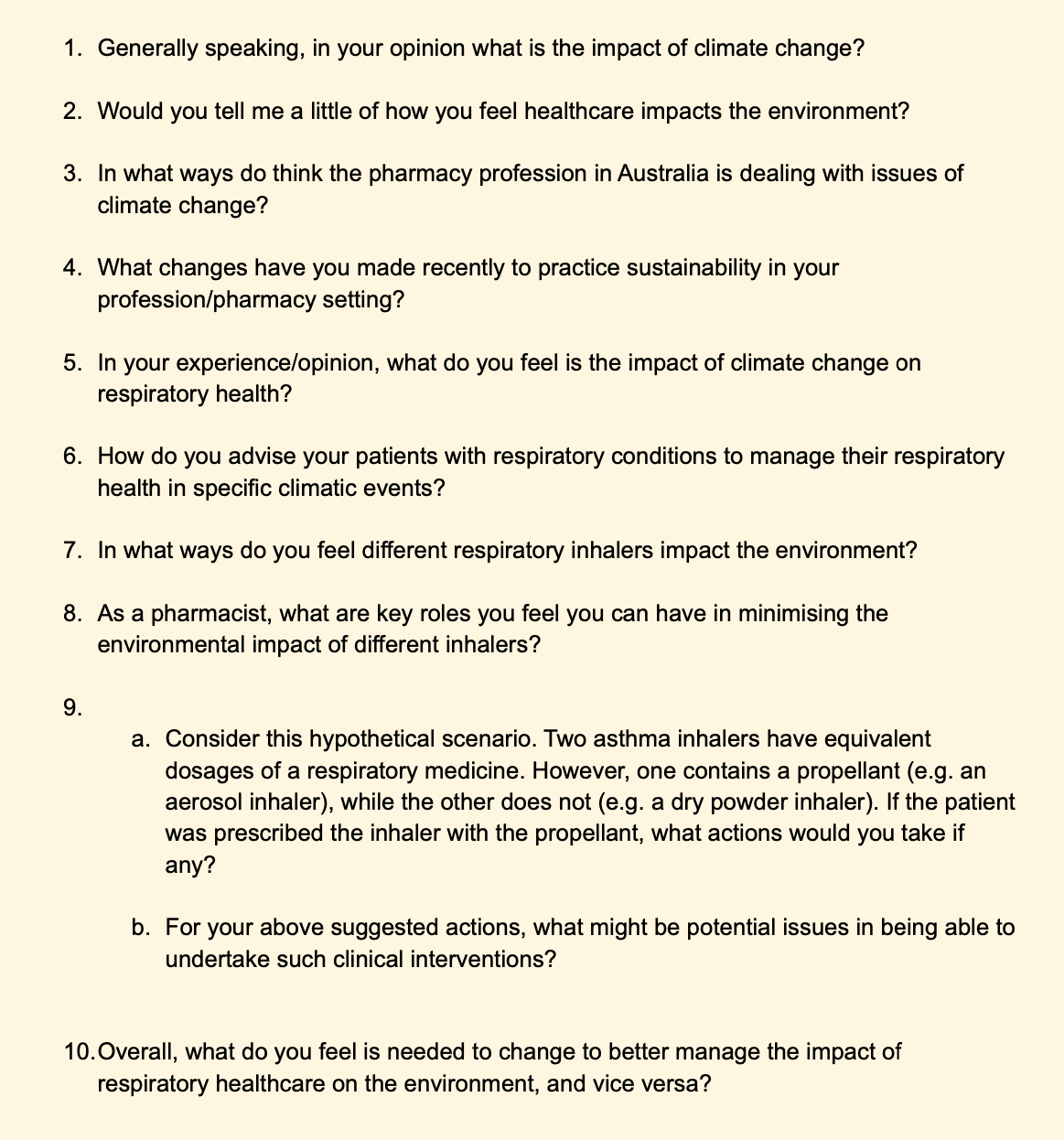
**

**Table 2.** Subthemes, codes and representative quotes for the theme ‘Environment considerations as an afterthought’

| **Subtheme** | **Code** | **Representative quote** |
| --- | --- | --- |
| Relevant yet minimal consideration | Climate change as important issue | “The peak bodies may have some stuff, but personally, I haven't come across them because it doesn't directly impact my daily practice much. Climate change, in my opinion, isn’t something I think about on a daily basis and doesn’t impact my practice much.” (participant #16, male, 6 years of experience) |
|  | Relevant in practice |  |
|  | Minimal relevance to practice |  |
| Patient health prioritisation | No value in environment-related discussions | “Who's training us to think about the impact the propellant is having on the environment, rather than the impact the inhaler will have on the patient's health? I would say no one. It's considered best practice to make sure that we know our patients know how to use their inhalers. We are taught from when we start our degree to when we become pharmacists with continuing our professional development that we need to make sure patient adherence is the best because data tells us that most patients are not using their inhalers correctly. Even with all this emphasis that we have on the importance of adherence and to correct technique. So if that's my number one thing then environmental impact is not at the top of the list.” (participant #32, male,8 years of experience)  “It doesn’t bother me too much, because for elderly patients it’s easier for them to get a new device rather than getting a refill and attaching it. And some patients are on multiple medications, you don’t want to add to the complexities.” (participant #16, male, 6 years of experience) |
|  | Focus during pre-registration training |  |
|  | Prioritisation of inhaler use which jeopardises environment |  |
| Uncoordinated efforts | Disconnected efforts in profession | “I think that there's not so much of an appetite. There's not so much of an incentive for small business owners or large businesses to involve themselves in that. The initiatives that I have seen have been, I guess you'd say, 'grassroots'. Someone at the pharmacy who cares about the issue pushing for... whether it be a recycling initiative or blister pack recycling. But I'd say that probably on the whole the pharmacy profession, from my perspective anyway, isn't doing much. I'm sure that there are things out there, but I'm just not…” (participant #2, male, 14 years of experience) |
|  | Primarily fuelled by personal motivation |  |

**Table 3.** Subthemes, codes and representative quotes for the theme ‘Linking environment to respiratory care

| **Subtheme** | **Code** | **Representative quote** |
| --- | --- | --- |
| Environment and respiratory advice | Primary role of patient management | “The better control of their respiratory health is gonna have an impact on the environment. So what we can do to change is to have a closer patient monitor of their respiratory health. Reviewing the patient more often. And the community pharmacists that have a role to play here. To talk to a patient about their management and how often they use their device. That can have an impact on the environment.” (participant #5, female, 5 years of experience)  “I would generally counsel the patients with asthma on always having a preventer and a reliever on hand. And using it if they need it, which I think would probably be the extent of my management of an emergency sort of talk that I would have with them.” (Interviewee #2, male, 14 years of experience)  “I really love the reusable soft mists inhalers, and not many patients know about this. But when they first came out, they were single-use and not reusable, and now they are reusable, and we can mark on the inhaler which definitely helps reduce waste. Also, certain inhalers now have counters. It encourages patients to use their whole inhaler completely until it’s finished.” (participant #14, female, 7 years of experience)  “Nothing really. It's all just the same as what it has been. I think the only thing has been where you can use the cartridges for the soft mist inhalers instead of using complete inhalers, trying to encourage people to do that where they can… But that's really the only thing, and it's such a minor thing. I don't know that it's having that much of an impact.” (participant #31, male, 38 years of experience) |
|  | Appropriate inhaler disposal |  |
|  | Promotion of reusable soft mist inhalers |  |
|  | Insignificant contribution of reusable soft mist inhalers |  |
| Barriers to change | Helplessness due to limited contributions | “I think we all try to do the right things. But I think one thing we definitely can't do a lot is- there's only so much that we can control when it comes to climate. A lot of times when we tell patients “Try to avoid allergens and things like that in allergic rhinitis.” But we know there's only so much you can avoid.” (participant #28, female, 7years of experience)  “I would say the resources available for training in this area are also limited other than asthma action plans and education of patients in inhaler techniques and appropriately manage their medication.” (participant #16, male, 6 years of experience)  “My grandma used to be on inhalers so I would always make sure to take out the ones that were empty and ensure she always had a new one to use. Then I’d think, what do I do with this? I guess just throw it in the bin. But it’s not just a medication box with a blister. It’s an actual metal canister. I think there should be better ways to dispose of this.” (participant #7, female, 12 years of experience)  “I think we looked into trying to organise a blister pack recycling bin for the pharmacy. But that involves money. And so we ultimately didn't get a final say in that.” (participant #2, male,14 years of experience) |
|  | Gap in knowledge around sustainability |  |
|  | Limited time and focussed responsibility |  |
|  | Physician authority |  |
|  | Organisational policies |  |

**Table 4.** Subthemes, codes and representative quotes for the theme ‘working towards sustainable practice’

| **Subtheme** | **Code** | **Representative quote** |
| --- | --- | --- |
| Desire for change | Domino effect | “From a community lens, how can we impact respiratory health on the climate at the moment? I don't think we could do a lot but we can advocate. Pharmacists have that role. We have a large voice. There's a lot of pharmacists. So we can advocate. We can be willing to accept change.” (participant #32, male, 8 years of experience)  “Through the projects that are currently running or in the works at the moment when it comes to the appropriate disposal of blister packs and working towards how exactly should we dispose of our MDIs. It’s more of an initiative. It's not in force that you need to do.” (participant #27, female, one and a half years of experience)  “I would talk to them about the fact that there's another product, right? The exact same ingredients but this is more environmentally friendly. And I'll show using a tester inhaler on how to use it and ask them are they comfortable with it. And if they are then I will encourage them to consider swapping over this particular medication, because one, they're still gonna get the same outcome therapy. Second, they're gonna be saving the environment. My action would be encouraging them towards the more environmentally friendly inhaler.” (participant #3, female, 5years of experience) |
|  | Exploring sustainable initiatives |  |
|  | Willingness to balance environment and patient |  |
| Need for leadership from larger organisations | Multi-stakeholder drive | “But if it’s accompanied by a logistical process, there would be more uptake. But this has to come from the government. I don’t see a pharmacist doing this.” (participant #16, male, 6 years of experience)  “That's why I support all these studies [participant was supporting the current research and similar studies]. Because if we don't speak about it, nothing gets done.” (participant #22, female,25 years of experience) |
|  | Policy mandates |  |
|  | Responsibility of pharmaceutical companies |  |
|  | Education provided by pharmaceutical professional bodies |  |
|  | Research institutions driving change |  |
|  | Incentives for services |  |
